# Supplementary material for: LC-SRM Combined With Machine Learning Enables Fast Identification and Quantification of Bacterial Pathogens in Urinary Tract Infections
Source: Mol Cell Proteomics. 2024 Aug 22;23(11):100832. doi: 10.1016/j.mcpro.2024.100832 (PMC11532907; doi:10.1016/j.mcpro.2024.100832)
Supplement: Supplemental Figures S1–S5 and Table S2 [file mmc10.pdf]

Supplemental data for :

LC-SRM combined with machine learning enables fast identification and quantification of bacterial pathogens in urinary tract infections

Clarisse Gotti[1-2], Florence Roux-Dalvai[1-2], Ève Bérubé[3], Antoine Lacombe-Rastoll[1-2], Mickaël Leclercq[1], Cristina C. Jacob[4], Maurice Boissinot[3], Claudia Martins[4], Neloni R. Wijeratne[4], Michel G. Bergeron[3] and Arnaud Droit[1-2]*

[1] Computational Biology Laboratory, CHU de Québec - Université Laval Research Center, Québec City, QC, Canada

[2] Proteomics Platform, CHU de Québec - Université Laval Research Center, Québec City, QC, Canada

[3] Centre de Recherche en Infectiologie de l'Université Laval, Axe Maladies Infectieuses et Immunitaires, Centre de Recherche du CHU de Québec-Université Laval, Québec City, QC, Canada

[4] Thermo Fisher Scientific, San Jose, CA, USA.

* To whom correspondence should be addressed : arnaud.droit@crchudequebec.ulaval.ca

Table of contents

List of figures

**Supplemental Figure S1**<br>Quality control	Page S-1
**Supplemental Figure S2**<br>Bacteria identification by machine learning on inoculated samples	Page S-2
**Supplemental Figure S3**<br>Linear regressions for all detected peptides of the 15 bacterial species	Page S-3
**Supplemental Figure S4**<br>Intensities of the 3 quantifier peptides of 4 bacterial species	Page S-4
**Supplemental Figure S5**<br>Quantification of UTI-positive patient urines	Page S-5

List of tables

**Supplemental Table S1**<br>Dataset and inoculations information	.xlsx file
**Supplemental Table S2**<br>Bacterial species culture conditions	Page S-6
**Supplemental Table S3**<br>SRM acquisition parameters and transition list	.xlsx file
**Supplemental Table S4**<br>Retention times of the 82 UTI signature peptide and 6 CytoC standards	.xlsx file
**Supplemental Table S5**<br>Quantification data for the *Transfer – Inoculations* dataset	.xlsx file
**Supplemental Table S6**<br>Linear regressions information for all the detected peptides for each bacterial species for the *Transfer – Inoculations* dataset	.xlsx file
**Supplemental Table S7**<br>Linear regressions, Pearson correlation coefficient, S/N ratio and LOQ for the 3 quantifier peptides per bacterial species for the *Transfer – Inoculations* dataset	.xlsx file

**Supplemental Table S8**<br>Quantification data for the *Transfer* – *Reproducibility* dataset	.xlsx file
**Supplemental Table S9**<br>Detailed results for the *Validation* – *Patients* dataset	.xlsx file
**Supplemental Table S10**<br>Detailed results for the *Validation* – *Inoculations* dataset	.xlsx file

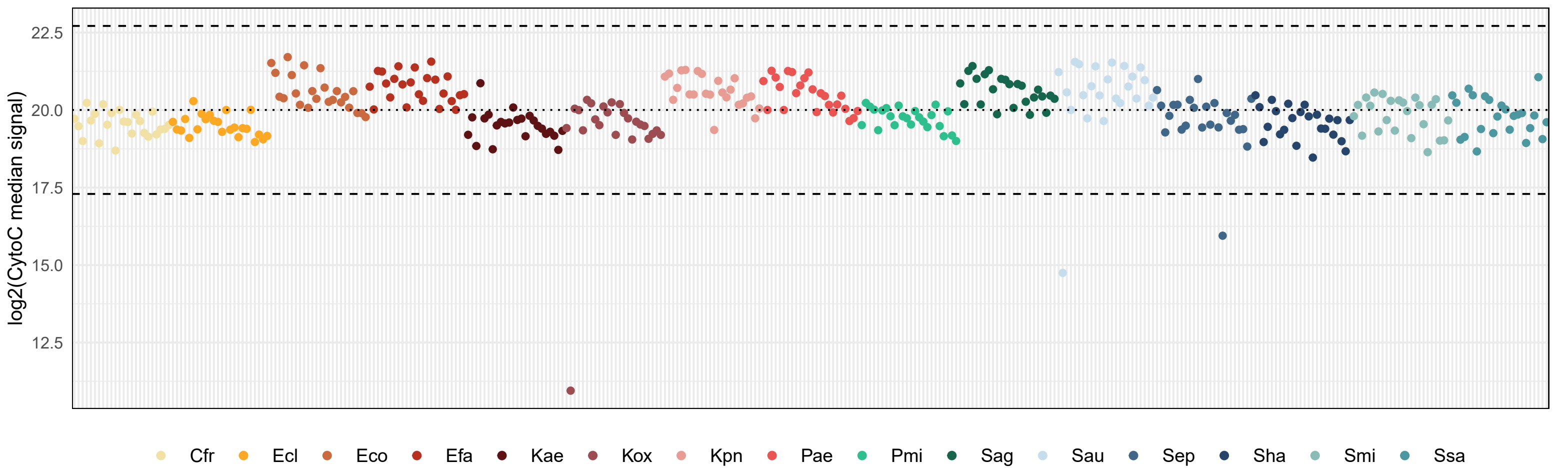

Supplemental Figure S1 : Quality control

Injections were controlled with CytoC standard peptides spiked in the sample just before injection. Average log2 area (dotted line) and +/- 3 standard deviation (dashed lines) were calculated over the 360 samples of the Transfer - Inoculations dataset.

A

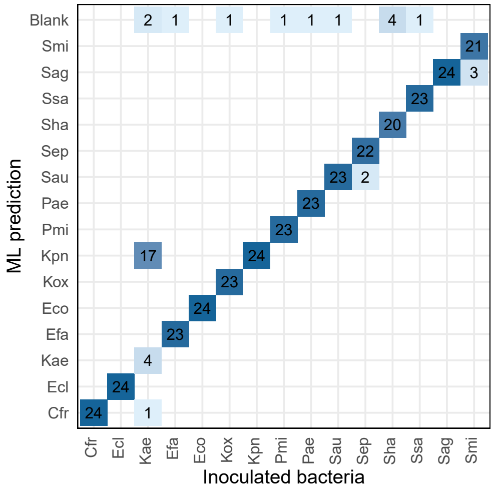

B

	DIA		SRM	
	Kae	Kpn	Kae	Kpn
SVEEILGK	0	8	24	24
STAETIVYSALETLAQR	0	8	18	24
AQYVLAEQVTR	0	7	13	23
FGGESVLAGSIIVR	10	0	0	0
VASLEGDVLGSYQHGAR	12	0	0	0

Supplemental Figure S2 : Bacteria identification by machine learning on inoculated samples

A) Confusion matrix of bacteria inoculated in healthy urines. Predictions by machine learning were done with the first generation model obtained from DIA data. B) Comparison of specific peptide signature to distinguish K. aerogenes (Kae) and K. pneumoniae (Kpn), in DIA and SRM data.

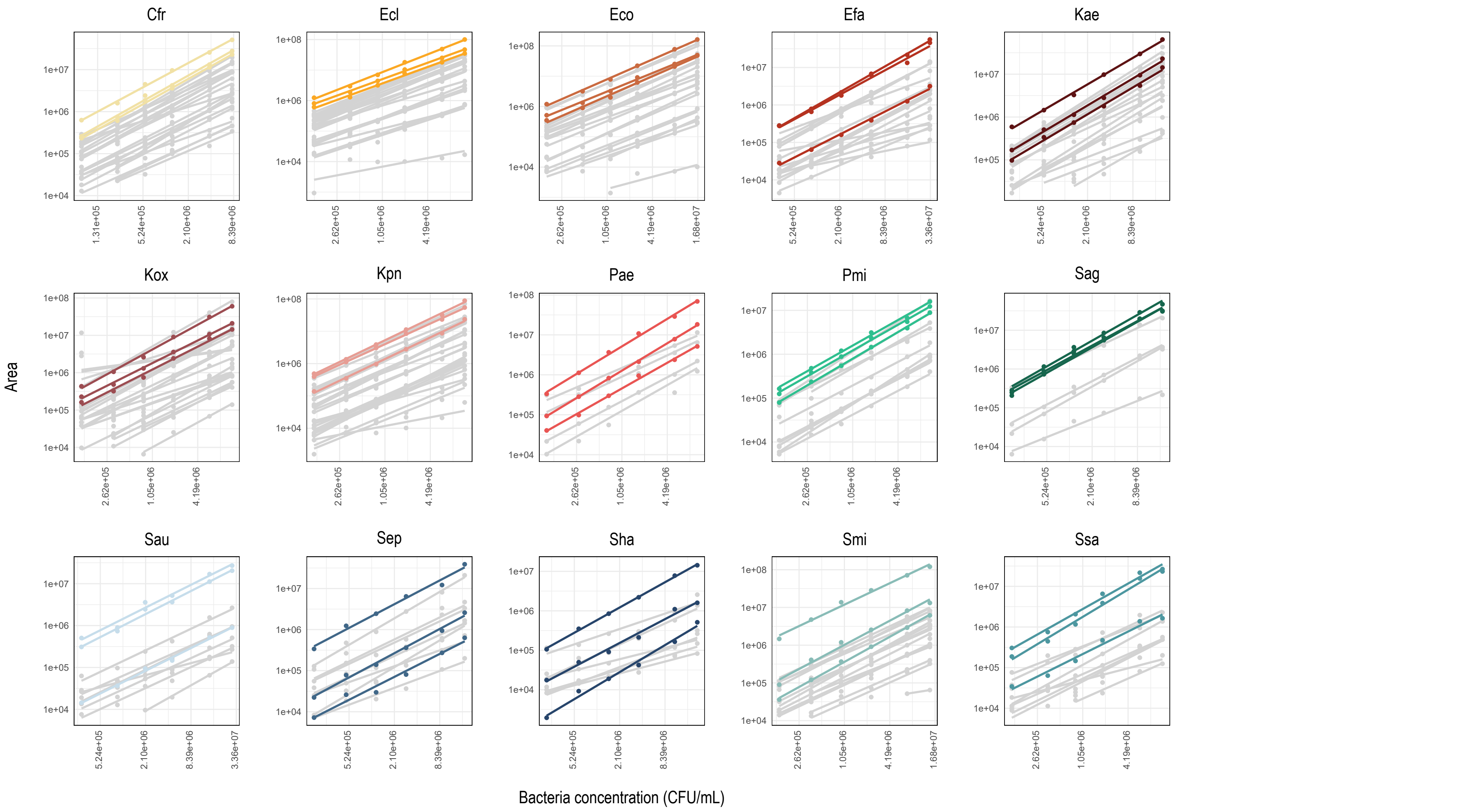

Supplemental Figure S3 : Linear regressions for all detected peptides of the 15 bacterial species
Linear regressions were calculated for all the detected peptides for each bacterial species, between the log_2 of the peptide peak areas and the log_{10} of the bacterial concentration inoculated in urine (CFU/mL). Peptides in colors are those selected for the quantification of unknown samples while those in grey (+ colored) will be only used for bacterial prediction by machine learning.

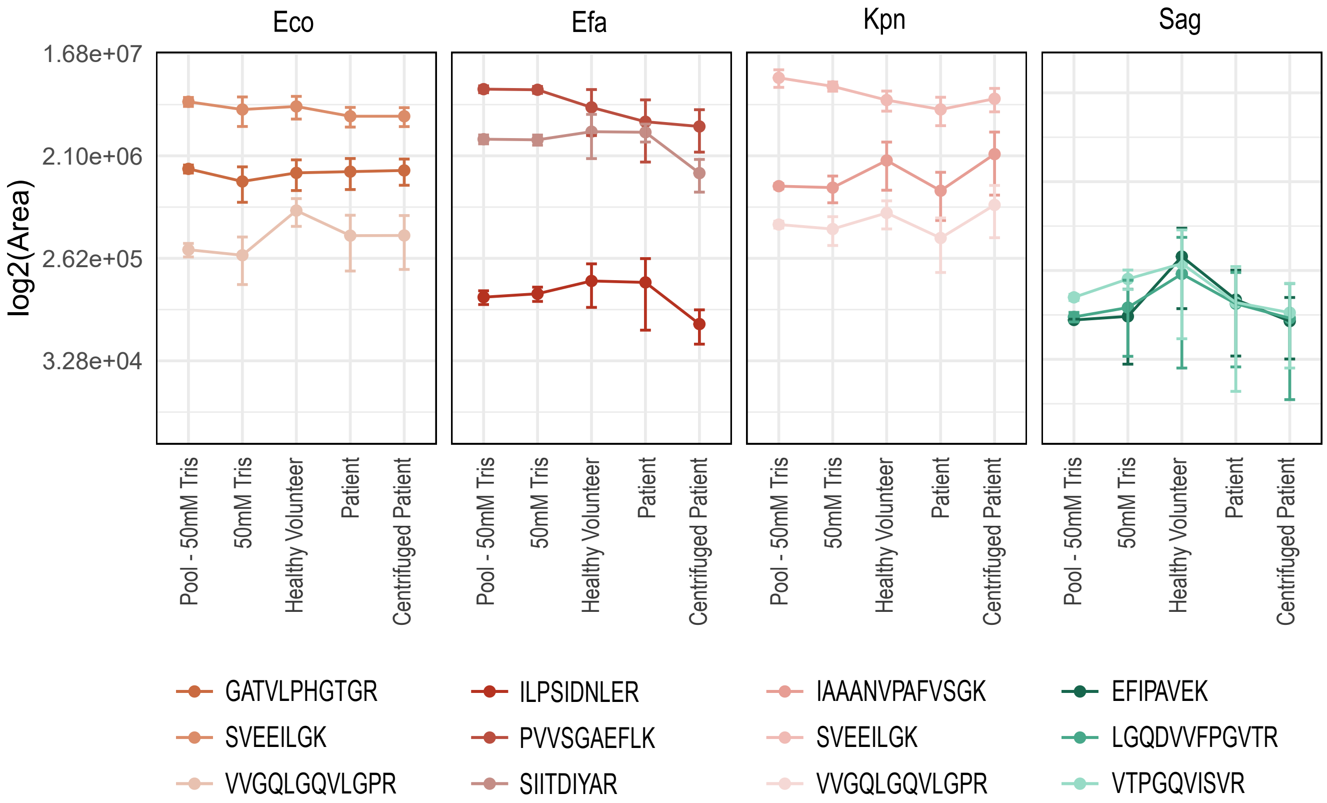

Supplemental Figure S4 : Intensities of the 3 quantifier peptides of 4 bacterial species

The log_2(Area) of each quantifier peptide was plotted for *E. coli* (Eco), *E. faecalis* (Efa), *K. pneumoniae* (Kpn) and *S. agalactiae* (Sag) for the 5 reproducibility testing methods : Pool - 50 mM Tris (injection); 50 mM Tris (technical); urines from healthy volunteers or from patients with or without a small centrifugation before bacterial isolation (biological).

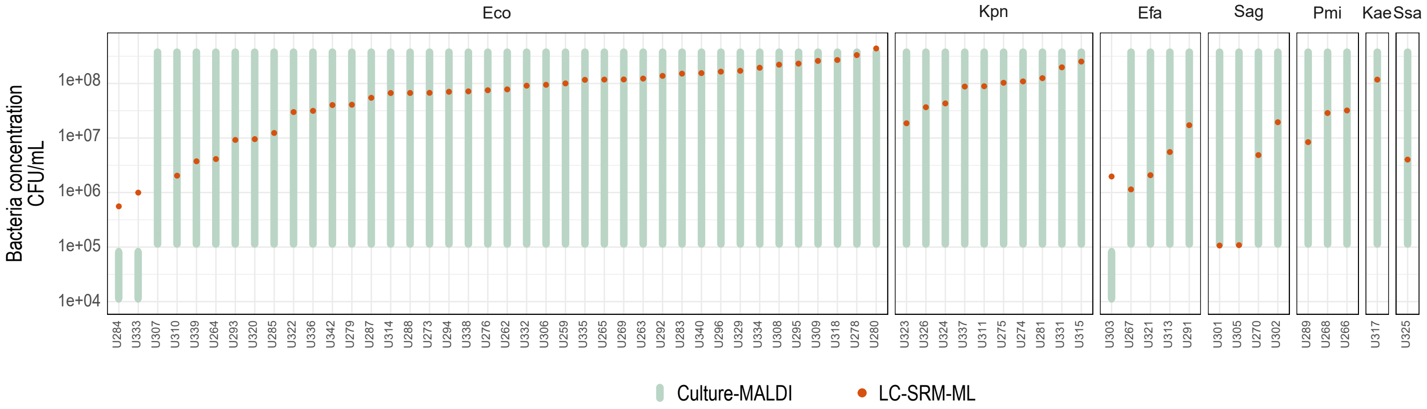

Supplemental Figure S5 : Quantification of UTI-positive patient urines
The 64 UTI-positive urines from patients whose machine learning prediction was in agreement with MALDI-TOF analysis were quantified by plate counting performed before MALDI-TOF analysis (green) or by LC-SRM (orange).

Bacterial species	Abbreviation	Strain	Culture condition	Gram-stain	Incubation time	Semi-log culture concentration (CFU/mL)
Citrobacter freundii	Cfr	CCRI-429	aerobe	negative	2h10	2,20E+08
Enterobacter cloacae	Ecl	CCRI-860	aerobe	negative	1h30	2,00E+08
Escherichia coli	Eco	CCRI-12923	aerobe	negative	1h50	4,70E+08
Enterococcus faecalis	Efa	CCRI-95	aerobe	positive	3h00	1,10E+09
Klebsiella aerogenes	Kae	CCRI-446	aerobe	negative	1h40	5,40E+08
Klebsiella oxytoca	Kox	CCRI-1109	aerobe	negative	2h00	5,20E+08
Klebsiella pneumoniae	Kpn	CCRI-563	aerobe	negative	1h40	3,90E+08
Pseudomonas aeruginosa	Pae	CCRI-691	aerobe	negative	3h00	9,30E+07
Proteus mirabilis	Pmi	CCRI-675	aerobe	negative	1h40	4,40E+08
Streptococcus agalactiae	Sag	CCRI-235	air + 5% CO_2	positive	2h30	2,30E+08
Staphylococcus aureus	Sau	CCRI-9443	aerobe	positive	1h50	3,10E+08
Staphylococcus epidermidis	Sep	CCRI-203	aerobe	positive	2h40	9,60E+07
Staphylococcus haemolyticus	Sha	CCRI-210	aerobe	positive	3h10	1,40E+08
Streptococcus mitis	Smi	CCRI-259	air + 5% CO_2	positive	4h45	1,30E+08
Staphylococcus saprophyticus	Ssa	CCRI-223	aerobe	positive	2h50	2,10E+08

Supplemental Table S2 : Bacterial species culture conditions
